# Long-Term Effect of Cover Crops on Species Abundance and Diversity of Weed Flora

**DOI:** 10.3390/plants9111506

**Published:** 2020-11-06

**Authors:** Alessia Restuccia, Aurelio Scavo, Sara Lombardo, Gaetano Pandino, Stefania Fontanazza, Umberto Anastasi, Cristina Abbate, Giovanni Mauromicale

**Affiliations:** Department of Agriculture, Food and Environment (Di3A), University of Catania, 95123 Catania, Italy; a.restuccia@unict.it (A.R.); saralomb@unict.it (S.L.); g.pandino@unict.it (G.P.); stefania.fontanazza@yahoo.it (S.F.); umberto.anastasi@unict.it (U.A.); cristina.abbate@unict.it (C.A.); g.mauromicale@unict.it (G.M.)

**Keywords:** cover crop, weed management, seed bank, weed associations, species richness, multivariate analysis, sustainability

## Abstract

Cover crops are gaining in popularity as an eco-friendly tool for weed control in organic and low-input agricultural systems. A 5-year study was carried out in a Mediterranean environment (Sicily, south Italy) to (1) quantify cover crop biomass production and (2) evaluate the effects on weed soil seed bank, aboveground biomass, species richness, species composition and associations between communities. Cover crop treatments included subterranean clover (*Trifolium subterraneum* L.) and spontaneous flora, both with and without burying dead mulch into the soil, compared to a conventional management treatment. Weed biomass was significantly reduced by subterranean clover, contrariwise to spontaneous flora, with season-dependent results. Cover crop biomass, which ranged from 44 to more than 290 g DW m^−2^, was negatively correlated to weed biomass. Moreover, subterranean clover decreased the size of the soil seed bank and species richness. Based on relative frequency, a low similarity was found between the conventional management and cover crop treatments. In addition, no significant differences in species composition across treatments were observed, whereas principal component analysis highlighted some associations. The results suggest that subterranean clover cover cropping is a good option for weed management in Mediterranean agroecosystems.

## 1. Introduction

Specialized orchards of the arid or semiarid regions of the Mediterranean basin are often characterized by low levels of soil organic matter and severe weed infestations, which need a frequent use of chemical inputs for their management [1]. In these agroecosystems, weeds represent the most serious constraint to agricultural production, causing serious yield losses due to their highly competitive capacity and allelopathic activity [2,3]. For many decades, they have been controlled almost exclusively through an irrational use of herbicides that, in addition to the negative effects on the environment, humans and animals [4,5], caused a significant reduction of biodiversity [6]. Low biodiversity in agroecosystems is associated not only to the development of a selective weed flora more difficult to manage, but also to a greater vulnerability to new invasive species [7]. Both weed abundance and diversity are closely influenced by agricultural practices, mainly soil tillage systems, crop rotation and fertilization [8], with a central role played also by environmental conditions [9,10]. The effects (positive or negative) of agronomic techniques on weed diversity are unclear and contradictory, depending on the specific conditions of field experiments, while conservation tillage systems are commonly reported to increase weed abundance [11]. Nowadays, given the increasing interest in limiting the dependence on herbicides, weed control in croplands is addressing to find ecologically-based practices (e.g., crop rotation, stale seedbed, cover cropping, mechanical and physical methods, etc.) under an integrated approach in a medium–long-term strategy [3]. The basic principle is that weeds are an integral part of the agroecosystem and, thus, they should be managed to reduce their harmful effects and increase benefits [12]. Integrated weed management systems are not absolute, but may vary in relation to the context-specific requirements and from year to year.

One of the most common eco-friendly practices, commonly adopted in organic and low-input agricultural systems, is cover cropping, which in the present study is going to include the techniques of mulching, intercropping and green manuring. Indeed, cover crops can be used as living mulches when intercropped between rows in herbaceous crops or on the whole field surface in tree crops, as well as dead mulches either on the soil surface or buried into the soil [3]. In both cases, they prevent weed germination and emergence physically by increasing the competition with weeds and chemically through allelopathic mechanisms [13]. In addition to the phytotoxic activity, cover crops are referred to increase soil fertility by reducing erosion and nutrient leaching, while improving the organic matter content, soil structure and microbial activities [14]. Among the high number of cover crops used in agroecosystems, the *Trifolium* genus and subterranean clover (*T. subterraneum*) in particular, play a key role in Mediterranean orchards thanks to N-fixation ability, rapid growth, rusticity, allelopathic activity and resistance to low radiation levels [15,16]. Subterranean clover originated in the Mediterranean basin, from where it spread throughout western Europe, northern Africa and other world regions with Mediterranean-type climates including Americas, New Zealand and mainly in southern Australia, where it is actually the major pasture legume [17]. It is a free-seeding annual legume, diploid (2n = 16) and predominantly self-pollinated, with remarkable geocarpism. Despite the dispute about the intraspecific taxonomy of *T. subterraneum*, recent genetic studies have confirmed the original classification provided by Katznelson and Morley [18], according to whom the species includes three subspecies with different ecological behavior: subsp. *subterraneum*, subsp. *yanninicum* and subsp. *brachycalycinum*.

In a recent study, Scavo et al. [1] demonstrated that *T. subterraneum* cover cropping significantly reduced the size of the weed seed bank, enhanced the amount of soil nitrogen bacteria and increased the levels of ammoniacal and nitric soil nitrogen. Developed as a continuation of the above-mentioned study, in this research we hypothesized that the observed changes in the potential weed flora (soil seed bank) could reflect on the real one in terms of abundance, richness and diversity, all key aspects for the development of an optimal integrated weed management strategy. Therefore, the objective of this work was to determine the long-term effect of *T. subterraneum* and spontaneous flora cover crops, with respect to a conventional management, on the aboveground weed populations and species composition in an apricot orchard.

## 2. Results

The real weed flora analysis showed that 38 weed species or genera were present in total throughout the study (Table 1), although most of them were not high frequent enough to be analyzed for principal component analysis (PCA). Seventeen botanical families were observed, the most representative of which was Asteraceae (32%), followed by Brassicaceae (10%) and Poaceae (8%). Concerning the life cycle, 55% were annuals, 29% perennials and 16% biennials. Moreover, 55% of weeds were therophytes and 39% hemicryptophytes, with only two geophytes: *Cirsum arvense* (L.) Scop. and *Convolvulus arvensis* L. (Table 1). Weed communities were dominated by dicotyledonous species (92%) and indifferent (26%) or spring–summer-germinating weeds.

### 2.1. Effect of Cover Cropping on Weed Diversity

ANOVA demonstrated that weed species richness varied in relation to both cover cropping and season, while their interaction was not significant (Table 2). The relationship between species richness and cover cropping was consistent at *p* ≤ 0.05, with only *Trifolium subterraneum* cover cropping leaving dead mulch on the soil surface (TCC-S) showing a significant reduction with respect to conventional apricot management (CM), contrary to *T. subterraneum* cover cropping burying dead mulch in the soil (TCC-B) that showed the highest value (10.2). Season had the greatest influence on the number of species (*p* ≤ 0.01). Overall, except for season III, weed species richness increased among years (+154% from season I to season V).

Jaccard and Sørensen’s indices were used to compare the similarity in terms of species composition between weed communities. Both showed very similar tendencies, with Sørensen’s coefficient always presenting higher values than Jaccard’s (Table 3). In total, following the trend of species richness, similarity increased across years (excluding season III), with values of +141% (J) and +98% (S) from the first to the last season. Regardless of season, SCC-S × SCC-B and TCC-S × TCC-B showed very high similarity (48.9% and 42.3% for J, 64.7% and 57.8% for S, respectively), while low values were determined between control and treatments (33.5% and 48% for TCC-S × CM, 30% and 44.3% for TCC-B × CM, respectively). The highest similarity was found between TCC-B and SCC-B in season V (92.9% and 96.3% for J and S, respectively), and the lowest one between TCC-B and SCC-S in season I (11.1% and 20% for J and S, respectively).

### 2.2. Effect of Cover Cropping on Weed Abundance

**Figure 1:**
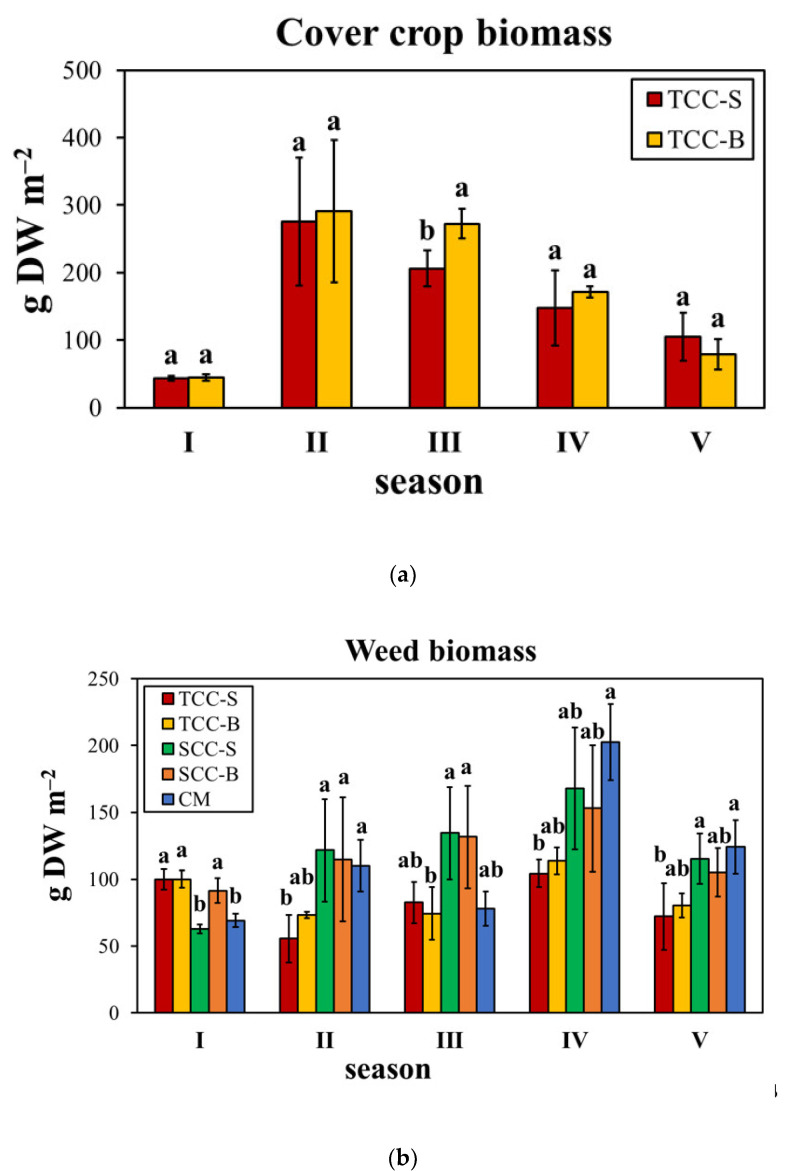
(**a**) Cover crop and (**b**) weed aboveground dry biomass production over five seasons in an apricot orchard. Bars are standard deviation (*n* = 4). Within each season, different letters indicate statistical significance at *p* ≤ 0.05 (Tukey’s HSD test). TCC-S: *Trifolium subterraneum* cover cropping leaving dead mulch on the soil surface; TCC-B: *T. subterraneum* cover cropping burying dead mulch in the soil; SCC-S: spontaneous flora cover cropping leaving dead mulch on the soil surface; SCC-B: spontaneous flora cover cropping burying dead mulch in the soil; CM: conventional apricot management. I: 2015/2016; II: 2016/2017; III: 2017/2018; IV: 2018/2019; V: 2019/2020.

The biomass production of subterranean clover did not statistically differ between TCC-S and TCC-B, although the incorporation of dead mulch into the soil resulted in a higher cover biomass for each season, except for the last one (Table 4). However, the effect of season was highly significant (*F*-Fisher = 63.6, *p* ≤ 0.001), with a general trend of season II > III > IV > V > I, suggesting a good establishment of the cover crop. ANOVA indicated no significance of the two-way interaction. On the contrary, weed biomass was significantly affected at *p* ≤ 0.001 by the interaction “cover cropping × season”, with 51.7% of the total variance explained by the latter factor. Overall, the biomass increased by 75.1% from season I to season IV and then decreased in the last season. Averaged over seasons, TCC-S and TCC-B decreased the weed biomass by 40.9% and 32.3%, respectively, as compared to CM (Table 4). The mean decrease highlighted by *T. subterraneum* cover cropping was marked in seasons IV (−5.5%), II (−70.6%) and V (−63%) (Figure 1). These seasons, with the exception of the third, were those with the major production of cover crop biomass. Indeed, a significant and negative relationship was found between these two parameters (*r* = −0.953, *p* = 0.0122), demonstrating that the lower the subterranean clover biomass is, the higher the weed biomass is. On the contrary, there was no correlation between species richness and weed biomass (*r* = −0.043, *p* = 0.944).

**Figure 2:**
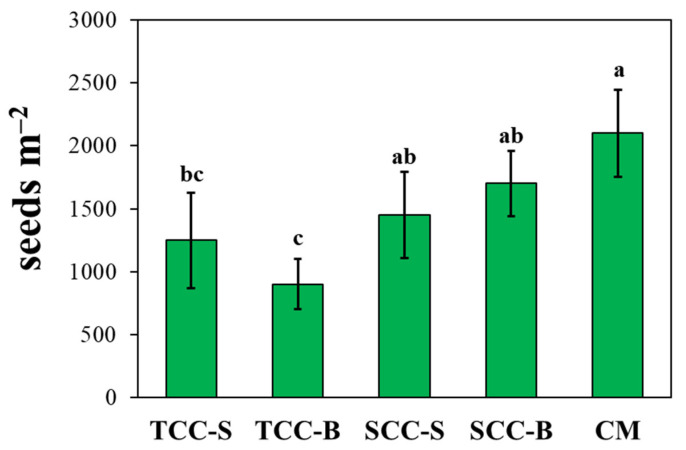
Cumulative effect on the soil weed seed bank after 5 years of different cover cropping systems. Bars are standard deviation (*n* = 4). Different letters indicate statistical significance at *p* ≤ 0.05 (Tukey’s HSD test). TCC-S: *Trifolium subterraneum* cover cropping leaving dead mulch on the soil surface; TCC-B: *T. subterraneum* cover cropping burying dead mulch in the soil; SCC-S: spontaneous flora cover cropping leaving dead mulch on the soil surface; SCC-B: spontaneous flora cover cropping burying dead mulch in the soil; CM: conventional apricot management.

Results on weed biomass were consistent with the potential flora. Indeed, all the cover cropping systems significantly lowered the number of weed seeds in the soil with respect to the conventional management (Figure 2). After 5-years, TCC-S and TCC-B had the lowest seed bank size, showing a reduction of 40.5% and 57%, respectively, compared to CM, in concordance with the aboveground weed biomass. However, the size of the soil seed bank was not correlated to the mean cover crop biomass (*r* = ‒0.827, *p* = 0.084), nor to the mean weed biomass (*r* = 0.767, *p* = 0.131). Anyway, despite the lack of significance, the seed bank decreased with increasing subterranean clover biomass; at the same time, average weed biomass was lower in TCC-S and TCC-B plots, where seed bank densities where the lowest.

### 2.3. Aboveground Weed Species Composition

Among the 38 taxa recorded throughout the 5-year period, only 11 weeds, predominantly annual seed-propagated species, showed a F ≥ 11% and a RF ≥ 3%: *Anagallis arvensis* L., *Beta vulgaris* L., *Cichorium intybus* L., *Ecballium elaterium* (L.) A. Rich., *Galium aparine* L., *Helminthotheca echioides* (L.) Holub, *Setaria verticillata* (L.) P. Beauv., *S. italica*, *Sinapis arvensis* L., *Sonchus asper* (L.) Hill and *Trigonella foenum-graecum* L. (Table 1). *Sonchus asper* was the most prominent weed species in all the seasons with mean values ranging from 8% to 31%, followed by *S. arvensis* with values in the range 15–22%. Moreover, averaged over seasons, this species showed the highest RF in TCC-S (12%), SCC-S (13%) and CM (9%) plots. Weed communities of TCC-B and SCC-B, instead, were dominated by *H. echioides* (F = 100, RF = 10%) and *E. elaterium* (F = 100, RF = 13%), respectively. However, the two-way ANOVA performed on RF data outlined that neither cover cropping nor season, except for some species, affected major weeds at *p* ≤ 0.05; for this reason, they have not been shown.

**Figure 3:**
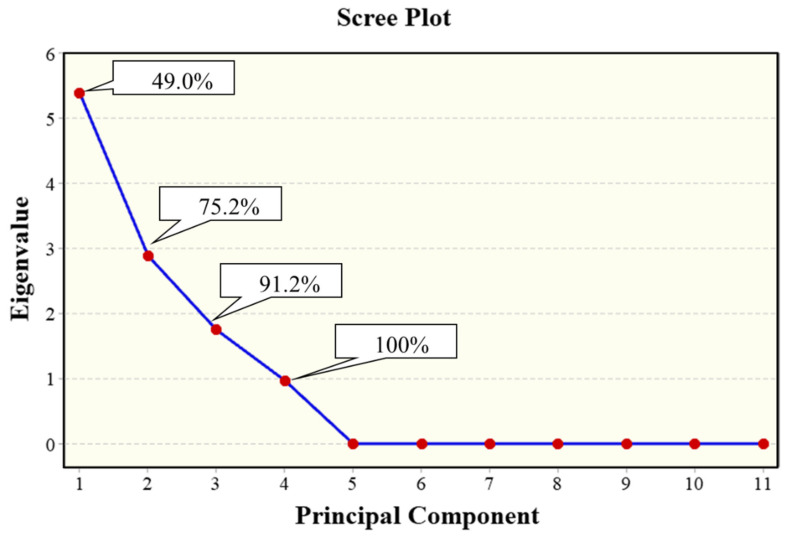
Scree plot of eigenvalues and cumulative variance of principal components from the correlation matrix.

**Figure 4:**
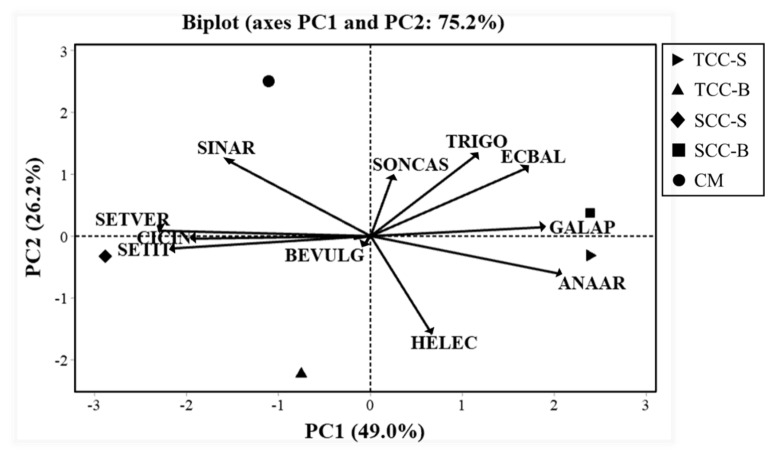
Principal components analysis ordination biplot from the correlation matrix with the 11 most frequent weed species, averaged over seasons, in different cover cropping treatments. Weeds: ANAAR (*Anagallis arvensis*); BEVULG (*Beta vulgaris*); CICIN (*Cichorium intybus*); ECBAL (*Ecballium elaterium*); GALAP (*Galium aparine*); HELEC (*Helminthotheca echioides*); SETVER (*Setaria viridis*); SETIT (*Setaria italica*); SINAR (*Sinapis arvensis*); SONCAS (*Sonchus asper*); TRIGO (*Trigonella foenum-graecum*). Treatments: TCC-S: *Trifolium subterraneum* cover cropping leaving dead mulch on the soil surface; TCC-B: *T. subterraneum* cover cropping burying dead mulch in the soil; SCC-S: spontaneous flora cover cropping leaving dead mulch on the soil surface; SCC-B: spontaneous flora cover cropping burying dead mulch in the soil; CM: conventional apricot management.

Among the 11 major weed species selected for PCA, the scree plot for standardized variables (correlation matrix) highlighted that only the first four PCs contributed to variance, while PC5-PC11 were insignificant (Figure 3). The cumulative variance explained by the first two eigenvalues together was 75.2%, which is an acceptable percentage for weed communities, thus suggesting a consideration of PC1 and PC2. The weeds *S. viridis*, *S. italica*, *A. arvensis*, *C. intybus* and *G. aparine* showed jointly the majority of variance (49%) in PC1; *E. echioides*, *T. foenum-graecum*, *S. arvensis* and *E. elaterium* added an additional 26% in PC2; *B. vulgaris* explained a further 16% of variance for PC3, while the eigenvector associated with PC4 corresponded to an eigenvalue <1, in which *S. asper* had the highest weight (Table 5). Table 5 showed also that PC1 was positively correlated to *G. aparine* and *A. arvensis* (right side of the biplot) and negatively by the two *Setaria* species and *C. intybus* (left side). A positive association was found between PC2 and *T. foenum-graecum*, *S. arvensis* and *E. elaterium* (top of biplot), and a negative one with *E. echioides* (bottom). Interesting associations were observed by PCA of weed species and cover cropping (Figure 4). *Setaria viridis*, *S. italica* and *C. intybus* were associated with SCC-S, whereas SCC-B was associated with *G. aparine* and TCC-S with *A. arvensis*. The other weeds were discriminated mainly along PC2, with a correlation observed between *S. arvensis* and CM, while TCC-B was not associated with any species, thus confirming a lower infestation in terms of weed biomass, soil seed bank and species composition. No relevant differences were observed between cover cropping systems in terms of botanical family, biological or ecophysiological groups, indicating no clear patterns of the weed flora.

## 3. Discussion

The present study aimed to evaluate the influence of 5 years of cover cropping, by subterranean clover and spontaneous flora, both buried and living dead mulches on the soil surface, on diversity and abundance of the real weed flora. In our previous research [1], we found a 70% reduction of the weed soil seed bank, compared to CM, after 3-years of *T. subterraneum* green manuring (TCC-B). Given that the real weed flora generally reflects the spectrum of the potential one, the effects on the emerged weeds were evaluated for a further two years on a medium–long-term period. We found that subterranean clover, in some seasons, significantly decreased the mean weed biomass up to 86%, contrariwise to spontaneous flora cover crop. The intensity of such a decrease was season-dependent, likely due to a combined effect of climatic conditions and cover crop biomass. In contrast with Moonen and Bàrberi [19], in our study cover crop biomass highly varied between the seasons from 44 to more than 290 g of DW m^‒2^. Weed biomass decrease caused by subterranean clover was higher in seasons when cover crop biomass was higher (seasons II, IV and V), except for season III. Our results are similar to those obtained by the study of Bàrberi and Mazzoncini [20], in which subterranean clover was found to reduce weed biomass from 21% to 67%, with a positive correlation between weed growth suppression and cover crop biomass and with seasonal effects. The results obtained on the real flora were corroborated by the effects on the soil seed bank, in which all the cover cropping systems decreased the number of weed seeds. TCC-S and TCC-B showed the highest weed suppressive ability after a further two years, although with a lower degree than the third year [1].

Weed suppressive ability of subterranean clover may be attributed to competitive or allelopathic effects, or even to a combination of them. *Trifolium subterraneum*, in fact, competes well with weeds thanks to its rapid growth, developed canopy, length of biological cycle and development of root system [21]. Generally, weed suppression increases with increasing cover crop biomass and cycle length, as found in the present study. Furthermore, subterranean clover is recognized as allelopathic species and allelochemicals responsible for such phytotoxic effects have been indicated as phenols and isoflavonoids [22]. These secondary metabolites can be directly exuded into the soil or released by decomposition of plant residues. Once present into the rhizosphere, allelochemicals interact with the complex of physical, chemical and biological soil characteristics, which altogether fix their availability [23]. Unfortunately, competitive and allelopathic effects are very difficult to distinguish in field experiments.

The emerged flora reflected the composition of the seed bank, given that weed communities were dominated by Asteraceae members, therophytes and annual spring–summer weeds. As previously observed on the seed bank [1], weed species richness was significantly affected by TCC-S, while, interestingly, TCC-B increased it, suggesting no clear influence of cover cropping. On the contrary, the effect of season was more noticeable, with a much higher number of weed species detected in season V, showing an increase in weed biodiversity. Conflicting reports have been provided by authors concerning the effects of cover crops on species richness. Ngouajio et al. [24], for example, observed no significant relationships, with results depending on cover crop type and season, while a reduction of weed density was found by Moonen and Bàrberi [19] using rye (*Secale cereale* L.) cover crop.

Since the contradictory results, many authors agree in not considering species richness as the only parameter to evaluate the herbicidal activity of cover crops. In this regard, the composition of weed communities plays a key role in shifting the phytotoxic effects. It should be pointed out, in fact, that the sensitivity of weed species to cover crop residue is highly variable, mainly depending on weed community structure. On one side, annual weeds with small seed sizes are more susceptible to surface residues than large seeded species, and on the other side, large seeds have a greater metabolic capacity for allelochemical detoxification [25]. In this study, the β-diversity indices of Jaccard and Sørensen were applied the compare the areas in terms of composition of the weed communities [26]. These indices are closely influenced by agronomic practices. Here, the highest similarity was found between spontaneous flora (SCC-S × SCC-B) and between subterranean clover (TCC-S × TCC-B) cover crop, often with values across seasons higher than 50%, at which an elevated similarity can be interpreted. Instead, a general low similarity was found between the conventional management and cover cropping systems. Therefore, it is reasonable to assume that *T. subterraneum* treatments (TCC-S and TCC-B) determined similar weed communities based on presence/absence, as well as spontaneous flora cover crops (SCC-S and SCC-B), both different with respect to CM. ANOVA performed on RF data of single species, however, pointed out any significant effect among treatments under study, demonstrating that weeds were able to establish independently of cover type and season. To overcome the complexity of weed data, species composition was studied by PCA on major weed species. In addition to a reduction in weed seed bank density and aboveground biomass, TCC-B did not show any association with weeds, contrariwise to SCC-S and SCC-B. No evident weed patterns emerged in this study, as observed also in the seed bank [1]. Overall, treatments were quite similar also with reference to frequency, botanical families, life cycle, biological and ecophysiological groups. The lack of consistent associations between cover crop and weeds has been reported in many other studies [19,27], since species composition can be influenced by abiotic and biotic factors. In the 9-year research study carried out by Shrestha et al. [28] on winter wheat and three beans, rye and maize cover crop were also indicated to have differential effects on weed densities, species composition and associations depending on crop type and interaction with agronomic management.

In conclusion, this research suggests that long-term changes in weed flora are linked to the soil seed bank. On one hand, the adoption of 5 years of cover cropping with subterranean clover was found to reduce not only the number of weed seeds in the soil, but also the aboveground weed biomass and the number of species, with significant variations by season. On the other hand, instead, no clear shifts in weed populations were observed. These results are very useful in view of reducing intensive tillage and the frequent application of herbicides, thus allowing multiple benefits for the environment. The benefits in using subterranean clover in Mediterranean agroecosystems are further increased considering its self-reseed capacity, N-fixation ability and high adaptability in such contexts [1]. Future studies may consider the evaluation of subterranean clover cover cropping in combination with other control techniques under an integrated weed management system, as well as a better knowledge of the mechanisms involved in its phytotoxicity.

## 4. Materials and Methods

### 4.1. Experimental Site and Set-Up

A field experiment was conducted over five growing seasons (from 2015/2016 to 2019/2020, hereafter named season I, II, III, IV and V) in an apricot (*Prunus armeniaca* L.) orchard sited in central Sicily (37°13′ N, 14°05′ E, 290 m a.s.l., Italy). The zone is subjected to a semiarid-Mediterranean climate, characterized by mean annual precipitations of ~500 mm, hot-rainless summers and mild winters. Based on the Rivas-Martinez bioclimatic classification, the area belongs to the thermo-Mediterranean inferior bioclimatic belt, with upper dry ombrotype. The experimental soil, Regosoil type according to the USDA soil taxonomy classification [29], at the beginning of the experiment presented an average soil texture of 25.7% sand, 30.6% silt and 43.7% clay, an average organic matter content of 1.9%, and an amount of 1.1‰, 13 mg kg^−1^ and 422 mg kg^−1^ of total nitrogen, assimilable P_2_O_5_ and exchangeable K_2_O, respectively, with pH 8.0.

For each growing season, the experiment was set-up in a randomized block design with four replicates (plot size = 10 × 8.7 m) including five treatments: four cover cropping systems compared to a conventional management (CM) as control following the standard commercial practices (−0.15 cm winter disc ploughing and three instances of shallow chopping per year for weed control). Cover cropping treatments were: (a) *T. subterraneum* cover cropping leaving dead mulch on the soil surface (TCC-S); (b) *T. subterraneum* cover cropping burying dead mulch in the soil (TCC-B); (c) spontaneous flora cover cropping leaving dead mulch on the soil surface (SCC-S), and (d) spontaneous flora cover cropping burying dead mulch in the soil (SCC-B). The experiment therefore included 20 plots and a net plot size of 1740 m^2^ (348 m^2^ per treatment), with a distance of 2 m between treatments.

The apricot orchard, composed by cv. Wonder and two pollinators (cvs. Pinkcot^®^ and Big Red^®^), was planted on January 2012 by using a 3.5 × 4.5 m arrangement. Subterranean clover cv. Seaton Park, a cheap and common Australian early-mid season genotype showing high adaptability in Mediterranean orchards [15], was hand-seeded on November 2015 at 2–3 cm depth with 2000 germinable seeds m^−2^. Detailed information about orchard management, fertilization, irrigation, weed and pest control were already reported in Scavo et al. [1]. Moreover, Table 6 summarizes the biological cycle of subterranean clover during the five growing seasons.

### 4.2. Monitoring, Sampling and Aboveground Biomass Determination

Monitoring was carried out by field scouting to visualize the weed spatial distribution, obtain a representative view of the weed flora and locate the sampling units. For each treatment, the sampling zone was chosen excluding the outer 3 m of each plot and the non-homogeneous areas. Within each zone, four permanent 1.0 m^2^ quadrats were randomly placed. The aboveground biomass of both weeds and subterranean clover was obtained by clipping in April for each season at soil surface from four 0.25 m^2^ patches per quadrat. In the laboratory, for TCC-S and TCC-B, cover crop biomass was separated from weed species and samples were dried at 55 °C in a forced-air oven up to constant weight for dry biomass determination. For the weed flora analysis, clipped weeds were identified according to Conti et al. [30] and grouped to botanical family and life-form category considering the Raunkiaer system; to obtain the total weed biomass per quadrat, the weed biomass was pooled at the quadrat level. The analysis of the weed soil seed bank was carried out in accordance with Scavo et al. [1]. In summary, soil samples were collected twice (April and September) per season at 10–15 cm depth along the diagonals of the central part of each sampling area and each soil sample was a composite of five soil cores per plot (each of 0.75 dm^3^). Then, the inert fraction (stones, pebbles, etc.) was hand-removed and a metal tube (Karcher, K 3500 model, Winnenden, Germany) with a removable cap fitted with steel mesh of 250 μm was used for seed extraction. Finally, the extracted fraction was placed inside Petri dishes for weed counts and identification by using a MS5 Leica stereomicroscope (Leica Microsystems, Wetzlar, Germany).

### 4.3. Weed Flora Analysis

Following Nkoa et al. [7] and Travlos et al. [31], the weed flora was analyzed by estimating species abundance and diversity. Abundance, describing the quantitative significance of a species in its habitat, was measured considering the total biomass of weeds (B), frequency (F) and relative frequency (RF):

(1) $$F\;\left(\%\right)=\;\left(\frac{\sum^{\;}Z_{i}}{n}\right)\;\times 100$$

(2) $$RF\;\left(\%\right)=\;\left(\frac{F_{i}}{\sum^{\;}F}\right)\;\times 100$$

where: ∑Z*_i_* = number of sampling units in which the species *i* occurred; *n* = total number of sampling units; F_i_ = absolute frequency of a species *i*; ∑F = sum of the absolute frequencies of all species. Despite needing destructive sampling, biomass, expressed as dry weight per unit area, is an accurate and objective index. F and RF are non-destructive indices reflecting the species’ spatial distribution across the sampled area and the changes over time.

In addition to species richness, i.e., the total number of species in each plot [19], the β-diversity was measured to estimate the species’ composition differences or similarity between communities. The β-diversity was calculated by using the Jaccard’s index of similarity (J) and the Sørensen’s coefficient index (S), computed as in Real and Vargas [32] and Nkoa et al. [7], respectively:

(3) $$J\;\left(\%\right)=\;\left(\frac{c}{a+b\;‒c\;}\right)\;\times 100$$

(4) $$S\;\left(\%\right)=\;\left(\frac{2c}{a+b}\right)\;\times 100$$

where: *a* = total number of species present only in one community; *b* = total number of species in the second community; *c* = total number of species common to each community. Both *J* and *S* are binary similarity coefficients based on presence/absence data.

### 4.4. Meteorological Trend

**Figure 5:**
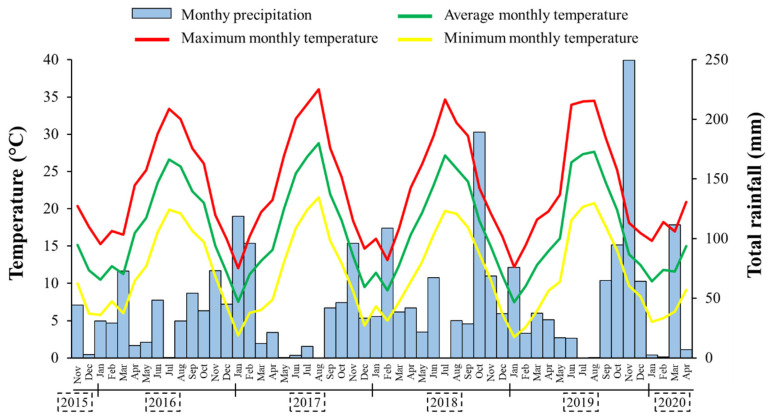
Total rainfall, maximum, average and minimum monthly temperatures distribution during the five cropping seasons.

A meteorological station (Mod. Multirecorder 2.40; ETG, Firenze, Italy) located at ~15 m on the experimental site was used to record rainfall and air temperatures every day during the five growing seasons, from November 2015 to April 2020 (Figure 5). Following the typical trend of the zone, summers were always particularly hot and dry, while most of rainfall fell in autumn. In particular, the sum of January 2016 (119 mm), October 2018 (189 mm) and November 2019 (250 mm) accounted for 22.1% of the total rainfall occurred in the whole experimental period. The years 2018 (668 mm) and 2019 (673 mm) experienced higher rainfall levels compared to the 30-year-period trend. The highest maximum temperature was recorded on August 2017 (36.1 °C), while the lowest minima temperatures were noted on January 2017 (3.0 °C) and 2019 (2.8 °C). The temperatures were always within the optimal range for *T. subterraneum* growth, since during the 5-year period, minimum temperatures only fall below 3.0 °C in season IV; the mean maximum temperatures were 25.7 °C in October (emergence) and 21.5 °C at flowering (April).

### 4.5. Statistical Analysis

Data about aboveground biomass of both subterranean clover and weeds, as well as species richness and soil seed bank, were analyzed through analysis of variance (ANOVA) by using the statistical software CoStat^®^ version 6.003 (CoHort Software, Monterey, CA, USA). Prior to ANOVA, the Bartlett’s and the Shapiro–Wilk tests were used to check for homoscedasticity and normality, respectively. Furthermore, to comply with the ANOVA basic assumptions, biomass and seed bank data were log_10_-transformed (untransformed data are reported), while species richness data did not show any violation and, therefore, they were not transformed. A factorial two-way ANOVA model with “cover cropping × season” as main factors was performed and means were separated with the Tukey’s HSD test at *p* ≤ 0.05. In some cases, one-way ANOVAs were applied. In accordance with Moonen and Bàrberi [19], correlations between soil seed bank size and *T. subterraneum* biomass, weed biomass, between species richness and weed biomass, and between cover crop and weed biomass, were calculated by using the Pearson Product Moment Correlation Coefficient (*r*) on mean values for these parameters.

To study the species composition and the interactions between cover cropping treatments and weed flora, a multivariate analysis was performed. Due to the high number of variables (weed species) composing the weed flora, the principal component analysis (PCA) was adopted to reduce the complex multivariate dataset in few orthogonal variables called principal components (PC) [33]. In particular, a PCA on the correlation matrix for 11 major variables (weeds with RF > 3%) was applied, considering the means for each “treatment × season” combination. Before PCA, all variables were standardized through arcsin √x (Bliss transformation), and then the results of the ordination were displayed on “distance” biplots deriving from the PCA by using the first two PCs [34]. The computer package Minitab^®^ version 16 (Minitab Inc., State College, PA, USA) was used to perform the PCA.

## Figures and Tables

**Table 1:** Botanical family, life cycle, ecophysiological (EG) and biological groups (BG), frequency (F) and relative frequency (RF) of weed population among 5 cropping systems and 5 seasons.

Weed Species	Botanical Family	Life Cycle	EG	BG	F (%) ^1^	RF (%) ^1^
*Avena* sp.	Poaceae	Annual	Sp	T	5.0	2.34
*Adonis annua* L. subsp. *cupaniana* (Guss.) Steinberg	Ranunculaceae	Annual	Sp	T	8.0	2.13
*Anagallis arvensis* L.	Primulaceae	Annual	Au-Wi	T	18.0	5.84
*Beta vulgaris* L.	Amaranthaceae	Perennial	Su	H	11.0	3.43
*Brassica rapa* L. subsp. *campestris* (L.) A.R. Clapham	Brassicaceae	Perennial	Sp-Su	H	1.2	0.17
*Capsella bursa-pastoris* (L.) Medik.	Brassicaceae	Biennial	Ind	H	1.0	0.18
*Chenopodium opulifolium* Schrad. ex W.D.J. Koch & Ziz	Chenopodiaceae	Annual	Su	T	1.0	0.44
*Chenopodium* sp.	Chenopodiaceae	Annual	Su	T	5.0	1.09
*Cichorium intybus* L.	Asteraceae	Perennial	Ind	H	14.0	3.23
*Cirsium arvense* (L.) Scop.	Asteraceae	Perennial	Su	G	2.0	1.0
*Convolvulus arvensis* L.	Convolvulaceae	Perennial	Ind	G	5.0	2.44
*Conyza canadensis* L.	Asteraceae	Annual	Sp-Su	T	3.0	1.20
*Daucus carota* L.	Apiaceae	Biennial	Sp-Su-Au	H	2.2	0.54
*Diplotaxis erucoides* (L.) DC.	Brassicaceae	Annual	Ind	T	8.0	2.54
*Dittrichia viscosa* (L.) Greuter subsp. *viscosa*	Asteraceae	Perennial	Au	H	1.0	0.22
*Ecballium elaterium* (L.) A. Rich.	Cucurbitaceae	Annual	Su	T	22.0	7.86
*Erigeron sumatrensis* Retz.	Asteraceae	Annual	Su	T	9.0	2.58
*Foeniculum vulgare* Mill.	Apiaceae	Perennial	Su	H	1.0	0.50
*Fumaria officinalis* L.	Fumariacee	Annual	Sp-Su-Au	T	10.2	2.52
*Galactites elegans* (All.) Soldano	Asteraceae	Biennial	Sp-Su	H	1.0	0.24
*Galium aparine* L.	Rubiaceae	Annual	Sp-Su-Au	T	15.8	3.68
*Glebionis coronaria* (L.) Spach	Asteraceae	Annual	Sp-Su	T	2.0	0.80
*Helminthotheca echioides* (L.) Holub	Asteraceae	Annual	Su-Au	T	48.6	13.16
*Hypochaeris radicata* L.	Asteraceae	Perennial	Sp	H	6.6	1.61
*Lamium amplexicaule* L.	Lamiaceae	Annual	Ind	T	2.0	0.36
*Malva sylvestris* L.	Malvaceae	Perennial	Ind	H	4.2	0.99
*Medicago polymorpha* L.	Fabaceae	Annual	Sp	T	2.2	0.35
*Papaver rhoeas* L.	Papaveraceae	Annual	Wi	T	9.2	2.39
*Reichardia picroides* (L.) Roth	Asteraceae	Perennial	Ind	H	9.8	1.77
*Setaria verticillata* (L.) P. Beauv.	Poaceae	Annual	Su	T	31.0	7.41
*Setaria italica* subsp. *viridis* (L.) Thell.	Poaceae	Annual	Su-Au	T	27.0	8.28
*Silene* sp.	Caryophyllaceae	Perennial	Sp-Su	H	3.8	0.64
*Sinapis arvensis* L.	Brassicaceae	Annual	Sp	T	51.0	14.79
*Sonchus asper* (L.) Hill	Asteraceae	Biennial	Ind	H	61.0	16.87
*Sonchus oleraceus* L.	Asteraceae	Biennial	Ind	H	11.0	2.76
*Stellaria media* (L.) Vill.	Caryophyllaceae	Biennial	Ind	H	1.0	0.40
*Trigonella foenum-graecum* L.	Fabaceae	Annual	Sp	T	11.0	3.59
*Vicia faba* L.	Fabaceae	Annual	Sp	T	5.0	1.25

Note: T: therophytes; H: hemicryptophytes; G: geophytes; Su, Au, Wi, Sp: summer, autumn, winter, spring species; In: indifferent species; ^1^ averaged over all treatments.

**Table 2:** Mean weed species richness found in 5 cropping systems and 5 seasons.

Treatment	No.	Season	No.
TCC-S	6.8 ^b^	I	4.4 ^b^
TCC-B	10.2 ^a^	II	9.0 ^a^
SCC-S	8.0 ^a^	III	7.4 ^ab^
SCC-B	8.0 ^a^	IV	9.6 ^a^
CM	8.6 ^a^	V	11.2 ^a^
*F*-test	*	*F*-test	**
SED ^1^	1.98	SED ^1^	1.37

Values within a column followed by the different letters are significant at *p* ≤ 0.05 (Tukey’s HSD test). SED: standard error of difference. ** and * indicate significance at *p* ≤ 0.01 and *p* ≤ 0.05, respectively. ^1^ 20 d.f. TCC-S: *Trifolium subterraneum* cover cropping leaving dead mulch on the soil surface; TCC-B: *T. subterraneum* cover cropping burying dead mulch in the soil; SCC-S: spontaneous flora cover cropping leaving dead mulch on the soil surface; SCC-B: spontaneous flora cover cropping burying dead mulch in the soil; CM: conventional apricot management; I: 2015/2016; II: 2016/2017; III: 2017/2018; IV: 2018/2019; V: 2019/2020.

**Table 3:** Jaccard’s (J, %) and Sørensen’s (S, %) similarity coefficients of β-diversity for a 5-cover cropping × 5 seasons system in an apricot orchard.

Treatments	I		II		III		IV		V	
	J	S	J	S	J	S	J	S	J	S
TCC-S × TCC-B	22.2	36.4	66.7	80.0	30.0	46.2	42.9	60.0	50.0	66.7
TCC-S × SCC-S	12.5	22.2	50.0	66.7	30.0	46.2	55.6	71.4	42.9	60.0
TCC-S × SCC-B	14.3	25.0	23.1	37.5	25.0	40.0	36.4	53.3	53.8	70.0
TCC-S × CM	12.5	22.2	50.0	66.7	16.7	28.6	42.9	60.0	45.5	62.5
TCC-B × SCC-S	11.1	20.0	66.7	80.0	16.7	28.6	33.3	50.0	50.0	66.7
TCC-B × SCC-B	28.6	44.4	38.5	55.6	23.1	37.5	23.5	38.1	92.9	96.3
TCC-B × CM	11.1	20.0	42.9	60.0	15.4	26.7	36.8	53.8	43.8	60.9
SCC-S × SCC-B	40.0	57.1	33.3	50.0	60.0	75.0	66.7	80.0	44.4	61.5
SCC-S × CM	14.3	25.0	50.0	66.7	66.7	80.0	42.9	60.0	37.5	54.5
SCC-B × CM	40.0	57.1	33.3	50.0	70.0	82.4	50.0	66.7	37.5	54.5

TCC-S: *Trifolium subterraneum* cover cropping leaving dead mulch on the soil surface; TCC-B: *T. subterraneum* cover cropping burying dead mulch in the soil; SCC-S: spontaneous flora cover cropping leaving dead mulch on the soil surface; SCC-B: spontaneous flora cover cropping burying dead mulch in the soil; CM: conventional apricot management; I: 2015/2016; II: 2016/2017; III: 2017/2018; IV: 2018/2019; V: 2019/2020.

**Table 4:** Effect of cover cropping (CC) and season (S) on aboveground dry biomass of *Trifolium subterraneum*, weeds and their sum (total) with analysis of variance (ANOVA, *F*-values).

Treatments		Aboveground Biomass (g DW m^‒2^)		
		*Trifolium Subterraneum*	Weeds	Total
CC	TCC-S	155.5 (43.1) ^a^	82.9 (15.2) ^b^	238.4 (40.7) ^a^
	TCC-B	171.7 (32.7) ^a^	88.3 (9.5) ^b^	260.0 (36.7) ^a^
	SCC-S	0.0	120.4 (28.0) ^a^	120.4 (28.0) ^b^
	SCC-B	0.0	119.2 (31.8) ^a^	119.2 (31.8) ^b^
	CM	0.0	116.8 (17.2) ^a^	116.8 (17.2) ^b^
S	I	44.4 (4.1) ^d^	84.7 (6.4) ^b^	102.5 (6.5) ^c^
	II	283.1 (99.9) ^a^	95.1 (24.8) ^b^	208.3 (35.6) ^a^
	III	239.2 (24.5) ^a^	100.3 (24.1) ^b^	195.8 (31.8) ^a^
	IV	159.4 (32.1) ^b^	148.3 (28.3) ^a^	212.0 (38.2) ^a^
	V	92.0 (29.1) ^c^	99.4 (18.2) ^b^	136.2 (19.7) ^b^
ANOVA				
	CC	0.4 NS	9.2 ***	64.1 ***
	S	63.6 ***	14.2 ***	31.6 ***
	CC × S	1.3 NS	4.0 ***	4.2 ***

Values are means with standard deviation (in brackets). Values within a column followed by different letters are significant at *p* ≤ 0.05 (Tukey’s HSD test). TCC-S: *Trifolium subterraneum* cover cropping leaving dead mulch on the soil surface; TCC-B: *T. subterraneum* cover cropping burying dead mulch in the soil; SCC-S: spontaneous flora cover cropping leaving dead mulch on the soil surface; SCC-B: spontaneous flora cover cropping burying dead mulch in the soil; CM: conventional apricot management; I: 2015/2016; II: 2016/2017; III: 2017/2018; IV: 2018/2019; V: 2019/2020. *** and NS indicate significance at *p* ≤ 0.001 and not significance, respectively.

**Table 5:** Eigenvectors defining the linear combination of variables (11 major weeds) and principal components from the correlation matrix (PC5‒PC11 were insignificant). The variables with the largest influence for each principal component are in bold.

Variable	PC1	PC2	PC3	PC4
ANAAR	**0.383**	−0.211	−0.167	0.182
BEVULG	−0.035	−0.015	**−0.752**	−0.056
CICIN	**−0.363**	−0.015	0.079	0.534
ECBAL	0.317	**0.382**	−0.056	−0.181
GALAP	**0.352**	0.050	0.319	0.388
HELEC	0.124	**−0.549**	−0.042	0.211
SETVER	**−0.430**	0.030	0.019	−0.027
SETIT	**−0.404**	−0.070	0.206	0.182
SINAR	−0.290	**0.426**	−0.094	−0.088
SONCAS	0.043	0.329	−0.410	**0.628**
TRIGO	0.215	**0.458**	0.273	0.121

ANAAR (*Anagallis arvensis*); BEVULG (*Beta vulgaris*); CICIN (*Cichorium intybus*); ECBAL (*Ecballium elaterium*); GALAP (*Galium aparine*); HELEC (*Helminthotheca echioides*); SETVER (*Setaria viridis*); SETIT (*Setaria italica*); SINAR (*Sinapis arvensis*); SONCAS (*Sonchus asper*); TRIGO (*Trigonella foenum-graecum*).

**Table 6:** Emergence, flowering and length of the biological cycle of subterranean clover among the five growing seasons under study.

Season	Emergence	Flowering	Length of the Biological Cycle ^1^
I	15 December 2015	22 May 2016	~200 days
II	22 November 2016	12 April 2017	~220 days
III	10 October 2017	29 April 2018	~250 days
IV	13 October 2018	26 April 2019	~240 days
V	5 November 2019	4 April 2020	~230 days

^1^ From the beginning of emergence until all the plants within a plot had completely dried up (first decade of July for all the seasons).

## References

[1] Scavo A., Restuccia A., Lombardo S., Fontanazza S., Abbate C., Pandino G., Anastasi U., Onofri A., Mauromicale G. (2020). Improving soil health, weed management and nitrogen dynamics by *Trifolium subterraneum* cover cropping. Agron. Sustain. Dev..

[2] Restuccia A., Lombardo S., Mauromicale G. (2019). Impact of a cultivation system upon the weed seedbank size and composition in a Mediterranean environment. Agriculture.

[3] Scavo A., Mauromicale G. (2020). Integrated weed management in herbaceous field crops. Agronomy.

[4] Scavo A., Restuccia A., Abbate C., Mauromicale G. (2019). Seeming field allelopathic activity of *Cynara cardunculus* L. reduces the soil weed seed bank. Agron. Sustain. Dev..

[5] Scavo A., Pandino G., Restuccia A., Lombardo S., Pesce G.R., Mauromicale G. (2019). Allelopathic potential of leaf aqueous extracts from *Cynara cardunculus* L. on the seedling growth of two cosmopolitan weed species. Ital. J. Agron..

[6] Landis D.A. (2017). Designing agricultural landscapes for biodiversity-based ecosystem services. Basic Appl. Ecol..

[7] Nkoa R., Owen M.D.K., Swanton C.J. (2015). Weed abundance, distribution, diversity, and community analyses. Weed Sci..

[8] Santín-Montanyá M.I., Martín-Lammerding D., Walter I., Zambrana E., Tenorio J.L. (2013). Effects of tillage, crop systems and fertilization on weed abundance and diversity in 4-year dry land winter wheat. Eur. J. Agron..

[9] Fried G., Norton L.R., Reboud X. (2008). Environmental and management factors determining weed species composition and diversity in France. Agric. Ecosyst. Environ..

[10] Gresta F., Cristaudo A., Onofri A., Restuccia A., Avola G. (2010). Germination response of four pasture species to temperature, light, and post-harvest period. Plant Biosyst..

[11] Bàrberi P. (2002). Weed management in organic agriculture: Are we addressing the right issues?. Weed Res..

[12] Jordan N., Vatovec C., Inderjit (2004). Agroecological benefits from weeds. Weed Biology and Management.

[13] Lemessa F., Wakjira M. (2014). Mechanisms of ecological weed management by cover cropping: A review. J. Biol. Sci..

[14] Gerhards R., Schappert A. (2020). Advancing cover cropping in temperate integrated weed management. Pest Manag. Sci..

[15] Mauromicale G., Occhipinti A., Mauro R. (2010). Selection of shade-adapted subterranean clover species for cover cropping in orchards. Agron. Sustain. Dev..

[16] Mauro R.P., Occhipinti A., Longo A.M.G., Mauromicale G. (2011). Effects of shading on chlorophyll content, chlorophyll fluorescence and photosynthesis of subterranean clover. J. Agron. Crop Sci..

[17] Zohary M., Heller D. (1984). The Genus Trifolium.

[18] Katznelson J., Morley F.H.W. (1965). A taxonomic revision of sect. *Calycomorphum* of the genus *Trifolium*. I. The geocarpic species. Isr. J. Bot..

[19] Moonen A.C., Bàrberi P. (2004). Size and composition of the weed seedbank after 7 years of different cover crop- maize management systems. Weed Res..

[20] Bàrberi P., Mazzoncini M. (2001). Changes in weed community composition as influenced by cover crop and management system in continuous corn. Weed Sci..

[21] Den Hollander N.G., Bastiaans L., Kropff M.J. (2007). Clover as a cover crop for weed suppression in an intercropping design: II. Competitive ability of several clover species. Eur. J. Agron..

[22] Liu Q., Xu R., Yan Z.Q., Jin H., Cui H.Y., Lu L.Q., Zhang D.H., Qin B. (2013). Phytotoxic allelochemicals from roots and root exudates of *Trifolium pratense*. J. Agric. Food Chem..

[23] Scavo A., Abbate C., Mauromicale G. (2019). Plant allelochemicals: Agronomic, nutritional and ecological relevance in the soil system. Plant Soil.

[24] Ngouajio M., McGiffen M.E., Hutchinson C.M. (2003). Effect of cover crop and management system on weed populations in lettuce. Crop Prot..

[25] Mirsky S.B., Ryan M.R., Teasdale J.R., Curran W.S., Reberg-Horton C.S., Spargo J.T., Wells M.S., Keene C.L., Moyer J.W. (2013). Overcoming weed management challenges in cover crop–based organic rotational no-till soybean production in the eastern United States. Weed Technol..

[26] Concenço G., de Farias P.M., Quintero N.F.A., Schreiber F., Galon L., Tomazi M., Moisinho I.S., Coradini M.C., Ceolin W.C., Andres A., Yousaf Z. (2017). Phytosociological surveys in weed science: Old concept, new approach. Plant Ecology-Traditional Approaches to Recent Trends.

[27] Swanton C.J., Shrestha A., Roy R.C., Ball-Coelho B.R., Knezevic S.Z. (1999). Effect of tillage systems, N, and cover crop on the composition of weed flora. Weed Sci..

[28] Shrestha A., Knezevic S.Z., Roy R.C., Ball-Coelho B.R., Swanton C.J. (2002). Effect of tillage, cover crop and crop rotation on the composition of weed flora in a sandy soil. Weed Res..

[29] Soil Survey Staff (1999). Soil Taxonomy: A Basic System of Soil Classification for Making and Interpreting Soil Surveys.

[30] Conti F., Abbate G., Alessandrini A., Blasi C. (2005). An Annotated Checklist of the Italian Vascular Flora.

[31] Travlos I.S., Cheimona N., Roussis I., Bilalis D.J. (2018). Weed-species abundance and diversity indices in relation to tillage systems and fertilization. Front. Environ. Sci..

[32] Real R., Vargas J.M. (1996). The probabilistic basis of Jaccard’s index of similarity. Syst. Biol..

[33] Deligios P.A., Carboni G., Farci R., Solinas S., Ledda L. (2019). The influence of herbicide underdosage on the composition and diversity of weeds in oilseed rape (*Brassica napus* L. var. *oleifera* DC) Mediterranean fields. Sustainability.

[34] Legendre P., Legendre L. (2012). Numerical Ecology.

